# Human-transmissible sarbecovirus ‘Khosta-2’ similar to SARS-CoV-2: potential global threat?

**DOI:** 10.1097/MS9.0000000000000486

**Published:** 2023-04-06

**Authors:** Ranjan K. Mohapatra, Snehasish Mishra, Ali A. Rabaan, Aroop Mohanty, Ranjit Sah

**Affiliations:** a Department of Chemistry, Government College of Engineering, Keonjhar; b School of Biotechnology, Campus, KIIT Deemed-to-be-University, Bhubaneswar, Odisha; c Department of Clinical Microbiology, All India Institute of Medical Sciences, Gorakhpur, Uttar Pradesh; d Department of Clinical Microbiology, Dr. D.Y. Patil Medical College, Hospital and Research Centre, Dr. D.Y. Patil Vidyapeeth; e Department of Public Health Dentistry, Dr. D.Y. Patil Dental College and Hospital, Dr. D.Y. Patil Vidyapeeth, Pune, Maharashtra, India; f Molecular Diagnostic Laboratory, Johns Hopkins Aramco Healthcare, Dhahran; g College of Medicine, Alfaisal University, Riyadh, Saudi Arabia; h Department of Public Health and Nutrition, The University of Haripur, Haripur, Pakistan; i Tribhuvan University Teaching Hospital, Institute of Medicine, Kathmandu, Nepal

Although hundreds of sarbecoviruses have been identified, mostly in Asian bats, but the majority do not infect human cells. Very recently, numerous sarbecoviruses are discovered in Russian horseshoe bats. The majority of these also do not infect human cells and were only distantly related to known human pathogens. However, a recently identified virus, Khosta-2, is found to be closely related to severe acute respiratory syndrome coronavirus 2 (SARS-CoV-2) that cause coronavirus disease 2019 (COVID-19). Researchers comprehend that it was capable of infecting humans and could be resistant to available vaccines as it spreads. A recently published research document reported that Khosta-2 infecting human cells and resistant to both the sera from SARS-CoV-2 vaccine recipients and SARS-CoV-2 monoclonal antibodies^1^. Khosta-2 and SARS-CoV-2 are both coronaviruses (CoV) belonging to the subclass sarbecovirus. Khosta-1 and Khosta-2 viruses found in Russian bats pose danger to humans. The origin of these viruses is mysterious. These unusual viruses (Khosta-1 and Khosta-2) were genetically from a viral lineage that is distinctly different from SARS-CoV and SARS-CoV-2. Researchers carefully investigated the viruses and suggested that they could infect human cells.

Khosta-1 demonstrated low human risk, whereas Khosta-2 verified likely troubling traits^1^. Seifert *et al.* 1 tested the receptor tropism and serological cross-reactivity of the receptor binding domain (RBD) in the two sarbecoviruses and found that the RBD of Khosta-2 was capable of using human angiotensin-converting enzyme 2 (ACE2) to facilitate cell entry as in SARS-CoV-2. Studies classify sarbecovirus RBDs into three clades: clade 1 (Asian bats) contains no deletions and binds to host receptor ACE2, clade 2 (Asian bats) contains two deletions and does not use ACE2, and clade 3 (African and European bats) contain 1 deletion and demonstrably could infect through primarily bat ACE2^2^–^5^. Several viruses of clade 4, identified in China in 2021, interact with ACE2^6^. Two clade 3 sarbecoviruses namely Khosta-1 (*Rhinolophus ferrumequinum*) and Khosta-2 (*Rhinolophus hipposideros*) were identified in 2020 in samples of Russian bat from the Sochi National Park^7^. The phylogeny of these viruses was closely related to another sarbecovirus BM48-31 (Bg08) reported from Bulgaria in 2008. Sarbecoviruses are circulating in wildlife beyond Asia including western Russia where from the Khosta-2 virus was reported. It may threaten global healthcare and the ongoing SARS-CoV-2 vaccination campaign.

There are reports of animals-to-human spillover events of sarbecoviruses leading to SARS-CoV and SARS-CoV-2 outbreaks. As discussed, the RBDs found in Khosta viruses are divergent from that of SARS-CoV and SARS-CoV-2. The viruses, particularly Khosta-2, seem lacking some genes that are supposedly involved in human pathogenesis. The potential risk to decipher is whether Khosta-2 can capably combine with SARS-CoV-2. The ability of SARS-CoV-2 to spillback from humans to the wildlife is revealed, but this trait is unclear in case of Khosta-2. Tests on the serum from COVID-19-vaccinated and the antibodies of omicron-infected individuals showed that it was ineffective in neutralizing Khosta-2. This demonstrates that a universal vaccine capable to protect against SARS-CoV-2, its emerging variants, and possible future sarbecoviruse strains, in general, is necessary, an urgent need to develop novel wide-spectrum sarbecovirus vaccine. Commercial vaccines against COVID-19 were designed with specific virus(es) in focus that need to be revisited and redesigned that could protect across all possible sarbecovirus threats.

The numerous gaps in understanding the host interactions in the transmission of the novel virus need to be urgently addressed. Active surveillance, investigation and monitoring efforts of the situation closely and in sync with all line-department agencies with active cross-border coordination are recommended to have a 360-degree approach to tackle the possible risk. In view of the overwhelmed medical system due to concurrent COVID-19 waves, risk assessment and control measures particularly in sensitive areas such as the natural habitat of such bat species may help counter future threat perceptions. Further, as spillover (animal-to-human) and spillback (human-to-animal) phenomena are on the rise as observed in recent times that threatens even the state-of-art healthcare system worldwide, the much sought-after ‘One Health’ strategy seems to have a robust and reliable answer that shall ensure a healthy world. With due coordination, direction and support by the WHO, healthcare experts around the world including veterinarians may lead from the front for greater success.

## Ethical approval

NA.

## Consent

NA.

## Sources of funding

No funding received.

## Author contribution

All the authors contributed significantly in developing the manuscript. R.K.M.: conceptualized the manuscript and wrote the first draft with input from A.A.R., S.M., R.S.: updated, reviewed, and edited the manuscript. All the authors have reviewed and approved the final draft for submission.

## Conflicts of interest disclosure

The authors declare that they have no financial conflict of interest with regard to the content of this report.

## Research registration unique identifying number (UIN)

NA.

## Guarantor

Ranjit Sah.

## Data availability statement

All data are within the manuscript.

## Provenance and peer review

Not commissioned, externally peer reviewed.
